# One‐year clinically important deterioration and long‐term clinical course in Japanese patients with COPD: a multicenter observational cohort study

**DOI:** 10.1186/s12890-021-01510-w

**Published:** 2021-05-12

**Authors:** Yuki Abe, Masaru Suzuki, Hironi Makita, Hirokazu Kimura, Kaoruko Shimizu, Satoshi Konno, Masaharu Nishimura

**Affiliations:** 1 Department of Respiratory Medicine, Faculty of Medicine and Graduate School of Medicine, Hokkaido University, Kita 15, Nishi 7, Kita-ku, Sapporo, 060-8638 Japan; 2 Hokkaido Medical Research Institute for Respiratory Diseases, Minami 3, Nishi 2, Chuo-ku, Sapporo, 060-0063 Japan

**Keywords:** Chronic obstructive pulmonary disease, Clinically important deterioration, Composite measures, Exacerbation, Mortality

## Abstract

**Background:**

Chronic obstructive pulmonary disease (COPD) is a heterogeneous disease with a complex progression of many clinical presentations, and clinically important deterioration (CID) has been proposed in the Western studies as a composite endpoint of disease progression. The aim of this study was to investigate the relationships between 1-year CID and the following long-term clinical outcomes in Japanese patients with COPD who have been reported to have different characteristics compared to the Westerners.

**Methods:**

Among Japanese patients with COPD enrolled in the Hokkaido COPD cohort study, 259 patients who did not drop out within the first year were analyzed in this study. Two definitions of CID were used. Definition 1 comprised ≥ 100 mL decrease in forced expiratory volume in 1 s (FEV_1_), ≥ 4-unit increase in St George’s Respiratory Questionnaire (SGRQ) score from baseline, or moderate or severe exacerbation. For Definition 2, the thresholds for the FEV_1_ and SGRQ score components were doubled. The presence of CID was evaluated within the first year from enrollment, and analyzed the association of the presence of CID with following 4-year risk of exacerbations and 9-year mortality.

**Results:**

Patients with CID using Definition 1, but not any single CID component, during the first year had a significantly worse mortality compared with those without CID. Patients with CID using Definition 2 showed a similar trend on mortality, and had a shorter exacerbation-free survival compared with those without CID.

**Conclusions:**

Adoption of CID is a beneficial and useful way for the assessment of long-term disease progression and clinical outcomes even in Japanese population with COPD. The definition of CID might be optimized according to the characteristics of COPD population and the observation period for CID.

## Background

Chronic obstructive pulmonary disease (COPD) is a heterogeneous condition with a complex progression of many clinical presentations, which vary in both their presence and severity [1]. The Global Initiative for Chronic Obstructive Lung Disease (GOLD) report describes that it is essential to personalize the assessment and clinical management of COPD, with the treatment goals of reducing the risk of future COPD exacerbations, minimizing disease activity, and preventing disease progression [2]. Quantifying disease activity and progression is a challenge in clinical studies and practice, and a more comprehensive approach to the management of COPD is needed. Therefore, a composite clinically important deterioration (CID) endpoint was developed to measure short-term worsening of disease status, comprising the following: lung function (≥ 100 mL decline in forced expiratory volume in 1 s [FEV_1_]), health status (≥ 4-unit increase in St George’s Respiratory Questionnaire [SGRQ]), and the incidence of moderate or severe exacerbation [3]. The occurrence of one or more of these events was defined as CID. The FEV_1_ and SGRQ thresholds were selected as the accepted minimum clinically important difference (MCID) for these outcomes [4, 5]. Several short-term (6- to 12-month) COPD studies have adopted this endpoint as a post hoc measure [6, 7], and associations between short-term CID and long-term outcomes have also been reported [8]. However, most of the participants in those studies were from Western countries, and it is still unclear whether this definition of CID is useful in diverse COPD populations.

Japanese patients with COPD have been reported to have different characteristics compared to those from Western countries; they are generally older and thinner, have lower SGRQ scores, and experience fewer exacerbations [9–11]. Therefore, we aimed to investigate whether the presence of any component of the composite CID over a 1-year period was associated with subsequent clinical outcomes (exacerbations and mortality) in Japanese patients with COPD. We used two different thresholds for the FEV_1_ and SGRQ components to define CID.

## Methods

### Study protocol and participants

The study is based on data from the Hokkaido COPD cohort study, which has previously been described in detail [12–16]. Japanese patients with COPD were enrolled from Hokkaido University Hospital (Sapporo, Japan) and nine affiliated hospitals between May 2003 and May 2005. All were aged over 40 years and were either current or former smokers with a smoking history of at least 10 pack-years. Participants diagnosed with clinical asthma were excluded by respiratory specialists. During the first year of follow-up, we reconfirmed the diagnosis based on the spirometry criteria of the GOLD guidelines [2]. A total of 279 patients with COPD (GOLD 1, 26 %; GOLD 2, 45 %; GOLD 3, 24 %; GOLD 4, 5 %) were followed up in the subsequent years. Until the 5th year, spirometry before and after bronchodilator inhalation was performed every 6 months. Health-related quality of life assessments with the SGRQ were conducted annually, and information on COPD exacerbations was collected monthly. Moderate exacerbation was defined as a worsening or new onset of either (1) two major symptoms (increased dyspnea, change in sputum purulence, or increased sputum volume) or (2) any major symptoms plus any minor symptoms (fever, increased cough, or wheezing) that required a prescription change. Severe exacerbation was defined by hospital admission. After the 5th year, spirometry after bronchodilator inhalation and diffusing capacity testing were conducted annually for those who agreed with the extension of the regular follow-up program until the 10th year. The majority of the participants continued to visit outpatient clinics for appropriate medical care, even if they dropped out of the regular follow-up program. Information on the fatal event was collected as previously described [16]. This study was conducted in accordance with the Declaration of Helsinki and approved by the ethics committee of Hokkaido University School of Medicine (med02-001). All participants provided written informed consent.

### Definitions and assessments of CID

Two definitions of CID were evaluated in this analysis. For Definition 1 (D1), we used the CID previously proposed by Singh et al. [3]. The CID was defined as any one of the following: (1) decrease ≥ 100 mL from baseline in post-bronchodilator FEV_1_, (2) increase ≥ 4 units in SGRQ score from baseline, and (3) incidence of moderate or severe exacerbation. In Definition 2 (D2), the thresholds for the FEV_1_ and SGRQ components were doubled, as follows: (1) decrease ≥ 200 mL from baseline in post-bronchodilator FEV_1_, (2) increase ≥ 8 units in SGRQ score from baseline; and (3) incidence of moderate or severe exacerbation. In each definition, participants who met at least one CID criteria within the first year of enrollment were classified as the CID1+ (D1) or CID2+ (D2) group, and those who did not, as the CID1- (D1) or CID2- (D2) group.

### Statistical analysis

Differences between the groups were analyzed using Fisher’s exact test for categorical variables, Student’s *t-*test for continuous parametric variables, and Mann–Whitney *U* test for continuous nonparametric variables. Exacerbation-free survival and mortality after the visit at 1 year were analyzed using the Kaplan–Meier method with the log-rank test and the multivariate Cox proportional hazards models. Covariates included in the Model 1 were age, sex, and smoking status at enrollment. Covariates included in the Model 2 were age, sex, smoking status, and post-bronchodilator FEV_1_ at enrollment. Mortality was analyzed separately for all-cause mortality and death from respiratory diseases (including respiratory failure, pneumonia, and lung abscess). Statistical significance was defined as p < 0.05. Statistical analyses were performed using JMP (SAS Institute Inc., Cary, NC, USA) and EZR version 1.54 (Saitama Medical Center, Jichi Medical University, Saitama, Japan), which is a graphical user interface for R software (The R Foundation for Statistical Computing, Vienna, Austria) [17].

## Results

**Fig. 1:**
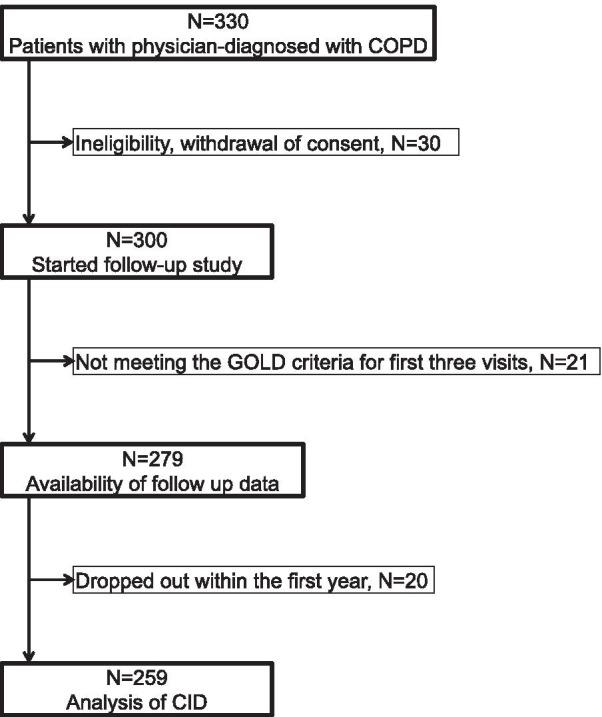
Flow-chart showing the study process

**Fig. 2:**
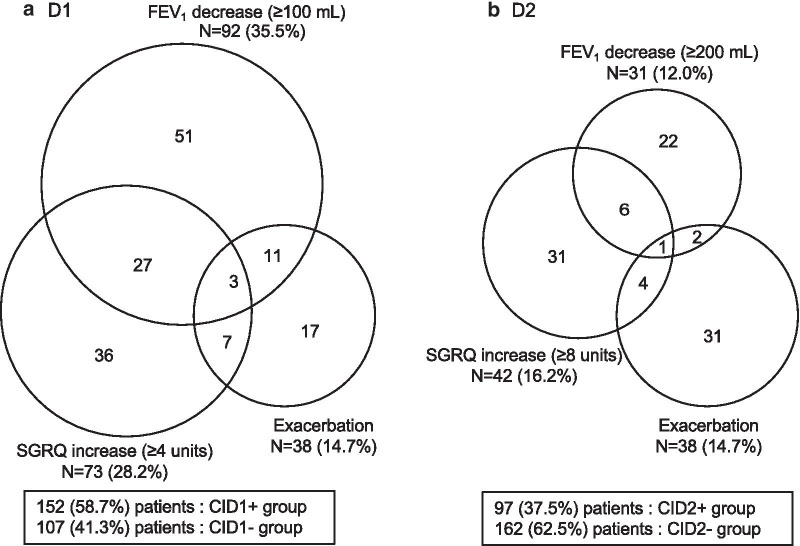
Individual components of the CID in the first year. Based on **a** D1 and **b** D2

Figure 1 shows the process of this study from registration to CID analysis. Of 279 patients with spirometry-confirmed COPD by the first year, 259 were eligible for this analysis, after the exclusion of 20 patients who dropped out within the first year. Venn diagrams showing the components of the CID based on each definition (D1 and D2) are shown in Fig. 2. In D1, 152 (58.7 %) patients were in the CID1 + group and 107 (41.3 %) patients were in the CID1- group. The most common cause of CID in the first year was deterioration in FEV_1_ (35.5 %), and the least was exacerbation (14.7 %). In D2, 97 (37.5 %) patients were in the CID2 + group and 162 (62.5 %) patients were in the CID2- group. Compared to D1, the positive rates of the three components were similar in D2 (deterioration in FEV_1_, 12.0 %; deterioration in SGRQ, 16.2 %; and exacerbation, 14.7 %).

**Fig. 3:**
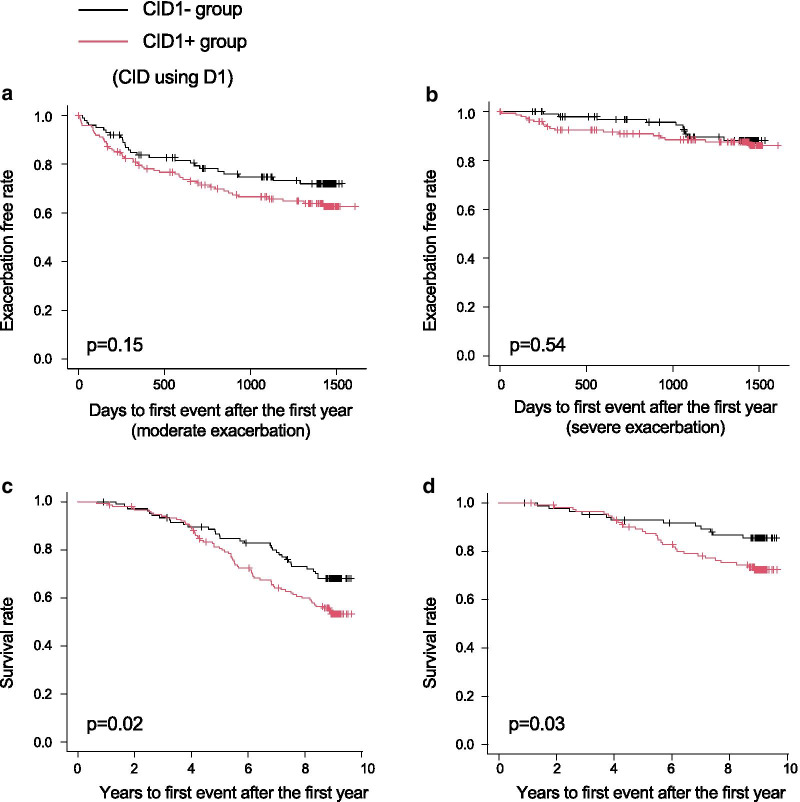
Kaplan–Meier curves according to CID using D1. **a** Moderate exacerbations. **b** Severe exacerbations. **c** All-cause mortality. **d** Death from respiratory diseases

**Fig. 4:**
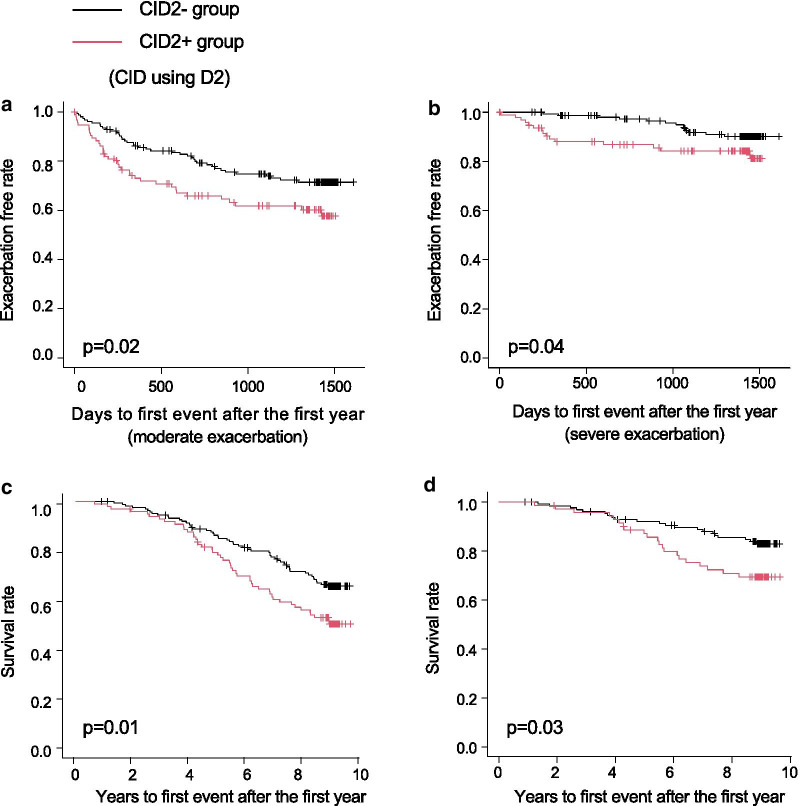
Kaplan–Meier curves according to CID using D2. **a** Moderate exacerbations. **b** Severe exacerbations. **c** All-cause mortality. **d** Death from respiratory diseases

Using D1, the post-bronchodilator FEV_1_/forced vital capacity (FVC) was significantly lower in the CID1 + group than in the CID1- group (Table 1). Although there were no associations between the two groups and the development of moderate or severe exacerbations (Fig. 3a, b), the CID1 + group had worse all-cause mortality and death from respiratory diseases than the CID1- group (*p* = 0.02, Fig. 3c; *p* = 0.03, Fig. 3d). Using D2, the post-bronchodilator FEV_1_ and FEV_1_/FVC were significantly lower in the CID2 + group than in the CID2- group (Table 1). Exacerbation-free survival was significantly shorter in the CID2 + group than in the CID2- group (moderate exacerbation, *p* = 0.02, Fig. 4a; severe exacerbation, *p* = 0.04, Fig. 4b). The CID2 + group had worse all-cause mortality and death from respiratory diseases than the CID2- group (*p* = 0.01, Fig. 4c; *p* = 0.03, Fig. 4d).

**Table 1:** Patient demographics and baseline characteristics, based on CID using D1 and D2

	All patients	CID1 + group	CID1 − group	P-value	CID2 + group	CID2 − group	P-value
	(n = 259)	(n = 152)	(n = 107)		(n = 97)	(n = 162)	
**Demographics**							
Age, years	69.6 ± 7.8	69.8 ± 7.5	69.4 ± 8.2	0.74	70.1 ± 7.2	69.4 ± 8.1	0.45
Female, N (%)	15 (5.8%)	7 (4.6%)	8 (7.5%)	0.42	4 (4.1%)	11 (6.8%)	0.43
Body mass index, kg/m^2^	22.2 ± 3.2	22.1 ± 3.3	22.5 ± 3.2	0.30	22.0 ± 3.2	22.4 ± 3.3	0.33
Current smoker, N (%)	73 (28.2%)	39 (25.7%)	34 (31.8%)	0.33	21 (21.7%)	52 (32.1%)	0.09
Pack-years	56.0 (43.0–78.0)	56.0 (43.0–75.8)	57.0 (43.0–79.5)	0.66	55.0 (42.0–73.5)	56.6 (43.5–82.0)	0.33
**Pulmonary function test**							
Post-BD FEV_1,_ L	1.7 ± 0.7	1.7 ± 0.7	1.8 ± 0.7	0.19	1.6 ± 0.6	1.8 ± 0.7	0.01
Post-BD FEV_1_, % predicted	64.5 ± 21.9	62.3 ± 21.8	67.8 ± 21.6	0.05	59.2 ± 20.9	67.8 ± 21.9	0.002
Post-BD FEV_1_/FVC, %	51.1 ± 12.5	49.6 ± 12.6	53.2 ± 12.1	0.02	48.2 ± 12.7	52.8 ± 12.2	0.003
DLco, % predicted	78.1 ± 25.0	76.2 ± 24.8	80.7 ± 25.2	0.16	77.3 ± 24.8	78.5 ± 25.2	0.70
Kco, % predicted	63.8 ± 24.3	62.6 ± 24.7	65.4 ± 23.7	0.37	63.3 ± 24.6	64.0 ± 24.2	0.82
**Patient-reported outcomes**							
Chronic bronchitis, N (%)	29 (11.2%)	16 (10.5%)	13 (12.2%)	0.69	13 (13.4%)	16 (9.9%)	0.42
mMRC dyspnea scale ≥ 2, N (%)	137 (52.9%)	84 (55.3%)	53 (49.5%)	0.38	55 (56.7%)	82 (50.6%)	0.37
SGRQ total score	31.8 ± 17.4	32.4 ± 17.4	31.1 ± 17.4	0.57	33.8 ± 17.3	30.7 ± 17.3	0.16
**CT emphysema score**	1.2 (0.7–2.0)	1.2 (0.8–2.2)	1.1 (0.6–2.0)	0.30	1.2 (0.7–2.0)	1.2 (0.7–2.2)	0.97
**Comorbidities**							
Any cardiovascular disease	58 (22.4%)	33 (21.7%)	25 (23.4%)	0.76	23 (23.7%)	35 (21.6%)	0.76
Ischemic heart disease	18 (7.0%)	10 (6.6%)	8 (7.5%)	0.81	6 (6.2%)	12 (7.4%)	0.80
Diabetes	12 (4.6%)	4 (2.6%)	8 (7.5%)	0.08	3 (3.1%)	9 (5.6%)	0.54

The multivariate Cox hazards proportional models showed that among single CID components, the exacerbation component was significantly associated with early onset of moderate exacerbation, and the SGRQ (≥ 8 units) component was associated with early onset of severe exacerbation and death from respiratory diseases, whereas other single components were not associated with future exacerbations. In addition, none of the single CID components were associated with all-cause mortality (Table 2). On the other hand, CID using D2, but not D1, was significantly associated with early onset of moderate exacerbation' (*p* = 0.01) and tended to relate to severe exacerbation (*p* = 0.06) (Table 2). CID using D1 was significantly associated with all-cause mortality and death from respiratory diseases(*p* = 0.047, 0.04), and CID using D2 tended to relate to all-cause mortality and death from respiratory diseases (*p* = 0.052, 0.0501). Furthermore, CID using D1 or D2 were tended to be associated with all-cause mortality after adjustments for post-bronchodilator FEV_1_ (Table 2).

**Table 2:** Hazard ratios of single CID events and composite CID events for CID + patients versus CID- patients

Risk factor	Moderate exacerbation		Severe exacerbation		All-cause mortality		Death from respiratory diseases	
	Hazard ratio	P-value	Hazard ratio	P-value	Hazard ratio	P-value	Hazard ratio	P-value
	(95 % CI)		(95 % CI)		(95 % CI)		(95 % CI)	
**Model 1**								
**Single CID components**								
FEV_1_ decrease (≥ 100 mL)	0.78 (0.47−1.26)	0.32	0.64 (0.25−1.44)	0.29	1.28 (0.84−1.91)	0.25	1.23 (0.63−2.29)	0.53
FEV_1_ decrease (≥ 200 mL)	0.59 (0.23−1.26)	0.19	0.98 (0.23−2.81)	0.97	1.39 (0.74−2.42)	0.29	1.77 (0.72−3.80)	0.20
SGRQ increase (≥ 4 units)	1.01 (0.61−1.62)	0.97	1.78 (0.82−3.74)	0.14	1.33 (0.88−1.99)	0.17	1.67 (0.88−3.08)	0.11
SGRQ increase (≥ 8 units)	1.28 (0.70−2.19)	0.41	2.88 (1.28−6.14)	0.01	1.39 (0.86−2.18)	0.17	2.28 (1.11−4.43)	0.03
Exacerbation	2.87 (1.64−4.83)	0.0004	1.09 (0.32−2.88)	0.87	1.17 (0.65−1.97)	0.59	1.00 (0.38−2.21)	1.00
**Composite CID**								
CID using D1	1.44 (0.90−2.34)	0.13	1.21 (0.57−2.73)	0.63	1.52 (1.006−2.34)	0.047	1.96 (1.02−4.02)	0.04
CID using D2	1.78 (1.12−2.81)	0.01	2.09 (0.98−4.53)	0.06	1.48 (0.997−2.20)	0.052	1.87 (0.9997−3.50)	0.0501
**Model 2**								
**Single CID components**								
FEV_1_ decrease (≥ 100 mL)	0.79 (0.48–1.26)	0.32	0.62 (0.24–1.40)	0.26	1.27 (0.84–1.90)	0.26	1.25 (0.64–2.33)	0.50
FEV_1_ decrease (≥ 200 mL)	0.60 (0.23–1.28)	0.21	1.03 (0.24–2.96)	0.96	1.40 (0.74–2.44)	0.28	1.79 (0.72–3.83)	0.19
SGRQ increase (≥ 4 units)	0.93 (0.56–1.50)	0.77	1.65 (0.76–3.49)	0.20	1.28 (0.84–1.92)	0.24	1.46 (0.76–2.74)	0.25
SGRQ increase (≥ 8 units)	1.10 (0.60–1.89)	0.75	2.59 (1.15–5.53)	0.02	1.33 (0.82–2.10)	0.24	1.99 (0.95–3.91)	0.07
Exacerbation	2.25 (1.28–3.82)	0.006	0.81 (0.23–2.18)	0.70	1.12 (0.62–1.89)	0.70	0.85 (0.32–1.93)	0.72
**Composite CID**								
CID using D1	1.35 (0.85–2.20)	0.21	1.07 (0.50–2.44)	0.86	1.48 (0.98–2.28)	0.07	1.80 (0.93–3.71)	0.08
CID using D2	1.54 (0.97–2.44)	0.07	1.79 (0.84–3.89)	0.13	1.44 (0.96–2.14)	0.08	1.66 (0.88–3.14)	0.12

CID, clinically important deterioration; CI, confidence interval; FEV_1_, forced expiratory volume in 1 s; SGRQ, St. George’s Respiratory Questionnaire; D1, Definition 1; D2, Definition 2

Model 1: Multivariate Cox proportional hazards models adjusted for age, sex, and smoking status. Model 2: Multivariate Cox proportional hazards models adjusted for age, sex, smoking status, and post-bronchodilator FEV_1_

At the beginning of the study, 13.9 % of all patients were using inhaled corticosteroids and 37.1 % were using long-acting beta-agonists, and after 5 and 10 years, 16.2 and 22.2 % were using inhaled corticosteroids and 39.3 and 44.4 % were using long-acting beta-agonists, with no major change during the follow-up period. None of the patients were using long-acting muscarinic antagonists at the start of the study because it is not clinically available in Japan. At baseline, 53.7 % of all patients were using short-acting muscarinic antagonists; after 5 and 10 years, 57.6 and 52.5 % were using muscarinic receptor antagonists, including long-acting muscarinic antagonists.

## Discussion

In this study, we evaluated whether CID status (a composite measure of early deterioration in COPD) assessed during the first year of observation could be used to predict long-term clinical outcomes of Japanese patients with COPD using data from the Hokkaido COPD cohort study. Two definitions of CID (D1 and D2) with different thresholds for FEV_1_ and SGRQ were used. We found that patients who experienced a CID event, but not any single component alone, in the first year had worse all-cause mortality than those who did not experience a CID event under both definitions. In addition, the presence of CID using D2, but not D1, was associated with an earlier onset of exacerbations.

CID is a composite endpoint that was recently introduced in a post-hoc analysis of COPD clinical trials [3, 18–20]. The decline in FEV_1_ over time has traditionally been an essential marker of disease progression and has been presented as a primary outcome in clinical trials. However, it is widely known that disease progression in COPD is not limited to a decline in FEV_1_, and that its progression can be monitored in many ways, including by monitoring changes in health status or physical activity over time, as well as the frequency of COPD exacerbations [21]. COPD is a multidimensional disease, and it has been reported that the correlations between changes in FEV_1_ and SGRQ [22] or exacerbations [23] were not strong. The composite CID endpoint was developed as a new approach to allow reliable monitoring of disease activity and progression using independent components and based on the principle of addressing different aspects of disease progression. Originally, CID consisted of lung function (≥ 100 mL decline in FEV_1_), health status (≥ 4-unit increase in SGRQ), and the incidence of moderate or severe exacerbation [3]. The FEV_1_ and SGRQ thresholds were selected from the established MCID [4, 5].

To evaluate the composite CID endpoint, it is important to know whether the composite CID is more useful than a single CID component as an outcome and whether it has prognostic ability in diverse COPD populations [24]. A post-hoc analysis of the 3-year TORCH and ECLIPSE studies showed that patients with CID had an increased risk of all-cause mortality compared with patients without CID after a CID assessment at 6 and 12 months, respectively [8]. It was also reported that patients with CID within the first 6 months of the UPLIFT study had worse outcomes for the remaining 42 months of the study [25]. However, the usefulness of CID in Japanese patients with COPD has not yet been clarified. In this study, we found that the presence of composite CID over a 1-year period was associated with subsequent exacerbations and better all-cause mortality than any single CID component (Table 2). This suggests that the composite CID endpoint would also be useful and beneficial among Japanese patients with COPD.

Recent reports on CID have explored other definitions in addition to the three-component CID definition as described above, including the COPD Assessment Test score [26] and the Transition Dyspnea Index [18]. However, there have been no studies that have used different thresholds for each CID component. When we adopted the original definition for each component of CID (i.e., D1), the positive rates of the three components were not uniform (Fig. 2a). On the other hand, when we used doubled thresholds for the FEV_1_ and SGRQ score components (D2), the positive rates of the three components were similar (Fig. 2b). In addition, CID using D2 was associated with a higher risk of exacerbations than CID using D1. We evaluated the CID over a 1-year period, which is longer than the TORCH [8] and UPLIFT [25] studies, in which CID was evaluated for 6 months. In addition, previous reports suggested that Japanese patients with COPD have less frequent exacerbations than patients in other countries [11]. Data from several global clinical trials also suggested that Japanese patients have a lower baseline SGRQ than patients in the rest of the world [9, 27, 28]. Therefore, the definition of CID might be optimized according to the characteristics of the COPD population and the period of evaluation in order to properly assess clinical outcomes using CID.

Although it is well known that the single CID components (worse pulmonary function, higher SGRQ score, and frequent exacerbations) are the risk factors for future exacerbation and mortality, none of the single CID components were associated with all-cause mortality, and only exacerbation component predicted moderate exacerbations in this study. The lack of significant association between single CID components and all-cause mortality may be due to a small sample size of this cohort, less frequent exacerbations, and better pulmonary function and health-related quality of life of Japanese patients with COPD [9, 11, 27, 28]. On the other hand, COPD exacerbation has a recurrent nature [29], and our previous study confirmed the recurrent nature of exacerbations even though the exacerbation frequency was low [14], which seemed to contribute to the association between single exacerbation component and future moderate exacerbations.

The use of CID is a useful approach for the assessment of long-term disease progression and clinical outcomes in patients with COPD, and thus potentially applicable for future large-scale randomized clinical trials. However, it remains uncertain whether the use of CID contributes to improved care of individual patients with COPD in practical clinical settings. It is well known that SGRQ scores reported by individual patients are sometimes unacceptably variable over time due to factors that may not necessarily be related to COPD itself. We have recently reported how variable pulmonary function parameters such as FEV_1_ are even among three acceptable trials [30]. The difference between the maximal and minimal FEV_1_ values among the acceptable trials was more than the MCID for FEV_1_ (100 mL) in as many as 14 % of the participants, regardless of the GOLD stages of airflow limitation.

This study has several limitations. First, the sample size was not as large as those in previous large-scale clinical studies. However, this study was carefully designed with high quality, the dropout rate was low, and the accurate mortality data were obtained for as long as 10 years. Second, the SGRQ was only assessed once a year, so it was not possible to assess the CID in a shorter period of time. However, the SGRQ is complex and difficult to assess frequently in clinical practice, and our results suggest that the assessment of CID on an annual basis may also play an important role in predicting subsequent long-term clinical outcomes. Third, the thresholds for each component of the CID have not been validated. The purpose of this study was not to find the most useful cutoff value for CID, but to examine the usefulness of CID in Japanese patients with COPD. Future large-scale studies may be needed to explore more appropriate thresholds for each component of CID.

## Conclusions

One-year deterioration assessed using a composite CID endpoint was associated with poor long-term clinical outcomes in Japanese patients with COPD. It was also suggested that the definition of CID should be optimized according to the characteristics of the COPD population and the period of evaluation. This concept of evaluating short-term changes across multiple endpoints may help identify patients at high risk for worsening of COPD earlier. Further research may be needed on the relationship between CID thresholds and long-term outcomes for future clinical trials.

## Data Availability

The datasets used and/or analyzed during the current study are available from the corresponding author on reasonable request.

## Abbreviations

COPD: chronic obstructive pulmonary disease
GOLD: Global Initiative for Chronic Obstructive Lung Disease
CID: Clinically important deterioration
FEV_1_: Forced expiratory volume in 1 s
SGRQ: St. George’s Respiratory Questionnaire
MCID: Minimum clinically important difference
D1: Definition 1
D2: Definition 2
FVC: Forced vital capacity
